# The Combination of MK-2206 and WZB117 Exerts a Synergistic Cytotoxic Effect Against Breast Cancer Cells

**DOI:** 10.3389/fphar.2019.01311

**Published:** 2019-11-06

**Authors:** Yu-Liang Li, Hao-Cheng Weng, Jui-Ling Hsu, Shu-Wha Lin, Jih-Hwa Guh, Lih-Ching Hsu

**Affiliations:** ^1^School of Pharmacy, National Taiwan University, Taipei, Taiwan; ^2^Department of Clinical Laboratory Sciences and Medical Biotechnology, College of Medicine, National Taiwan University, Taipei, Taiwan

**Keywords:** MK-2206, WZB117, Akt, GLUT1, DNA damage and repair, breast cancer

## Abstract

Breast cancer is the most commonly diagnosed cancer and the second leading cause of cancer death in women. Hormone receptor-positive breast cancer is usually subjected to hormone therapy, while triple-negative breast cancer is more formidable and poses a therapeutic challenge. Glucose transporters are potential targets for the development of anticancer drugs. In search of anticancer agents whose effect could be enhanced by a GLUT1 inhibitor WZB117, we found that MK-2206, a potent allosteric Akt inhibitor, when combined with WZB117, showed a synergistic effect on growth inhibition and apoptosis induction in breast cancer cells, including ER(+) MCF-7 cells and triple-negative MDA-MB-231 cells. The combination index values at 50% growth inhibition were 0.45 and 0.21, respectively. Mechanism studies revealed that MK-2206 and WZB117 exert a synergistic cytotoxic effect in both MCF-7 and MDA-MB-231 breast cancer cells by inhibiting Akt phosphorylation and inducing DNA damage. The combination may also compromise DNA damage repair and ultimately lead to apoptosis. Our findings suggest that the combination of Akt inhibitors and GLUT1 inhibitors could be a novel strategy to combat breast cancer.

## Introduction

Breast cancer is the most commonly diagnosed and the second leading cause of cancer death in women according to the survey of the American Cancer Society (Siegel et al., 2019). Hormonal therapy and targeted therapy are available for estrogen receptor-positive and HER2-positive breast cancers; however, triple-negative breast cancers (15–20% of total breast cancers) are more difficult to manage and mainly rely on chemotherapy. Drug resistance often develops following drug treatment; therefore, combination therapy is a better approach not only to improve therapeutic efficacy but may also circumvent drug resistance. The combination of anthracyclines such as doxorubicin and taxanes such as paclitaxel is commonly used for breast cancer treatment. However, increasing resistance to these agents has been observed (Dean-Colomb and Esteva, 2008). Thus, new combination strategies are needed.

Otto Warburg found long ago that glucose metabolism in cancer cells is different from that in normal cells. Cancer cells tend to utilize the glycolysis pathway even when oxygen supply is sufficient. This phenomenon is called “aerobic glycolysis,” also known as the Warburg effect (Vander Heiden et al., 2009). Since cancer cells have a higher glycolysis rate, blocking this energy production pathway seems reasonable to selectively kill cancer cells. Producing energy from glycolysis is not as efficient as from oxidative phosphorylation. Therefore, cancer cells need more glucose to support the higher glycolysis rate. Elevated expression of glucose transporters, especially GLUT1, has been found in many cancers (Ganapathy-Kanniappan and Geschwind, 2013; Qian et al., 2014).

WZB117, a synthetic small-molecule GLUT1 inhibitor, has been demonstrated to have anticancer activity against non-small cell lung cancer cells both *in vitro* and *in vivo*. It was reported that WZB117 downregulated the levels of GLUT1 protein, intracellular ATP, and glycolytic enzymes and upregulated the phosphorylation of ATP-sensing enzyme AMPK, leading to cell cycle arrest, senescence, and necrosis in A549 non-small cell lung cancer cells. WZB117 also displayed a synergistic anticancer effect when combined with cisplatin or paclitaxel in A549 and MCF-7 cells (Liu et al., 2012). A higher glycolysis rate in tumor cells may also confer resistance to chemotherapeutics (Ganapathy-Kanniappan and Geschwind, 2013). It has also been reported that WZB117 can overcome 5-FU chemoresistance in colorectal cancers and radioresistance in breast cancers (Liu et al., 2014; Zhao et al., 2016).

The PI3K/Akt signaling pathway plays an important role in tumor progression. *PIK3CA* and *Akt* mutations, and loss of PTEN, a negative regulator of the PI3K/Akt pathway, have been frequently found in cancers and lead to overactivation of this pathway which is associated with cancer cell growth, survival, and metabolism (Liu et al., 2009). Since the PI3K/Akt pathway is critical for cancer cells, this cascade provides good drug targets. Moreover, constitutive activation of this pathway is also associated with resistance to chemotherapy (West et al., 2002). Thus, targeting the PI3K/Akt pathway is a promising strategy for cancer therapy as well as overcoming chemoresistance.

Akt can activate its downstream serine/threonine kinase mammalian target of rapamycin (mTOR), which then phosphorylates p70 ribosomal protein kinase (p70S6K) and 4E-binding protein 1 (4E-BP1), leading to initiation of protein synthesis. Deregulation of this pathway is reported to enhance cell survival, increase proliferation, suppress apoptosis, and contribute to neoplastic transformation (West et al., 2002; Liu et al., 2009). MK-2206 is a potent allosteric Akt inhibitor in clinical development for the treatment of solid cancers. It has been reported that MK-2206 has antitumor activity both *in vitro* and *in vivo* either alone or in combination with molecular targeted agents such as erlotinib and lapatinib or cytotoxic agents such as docetaxel and carboplatin (Hirai et al., 2010). Mechanisms underlying cytotoxicity of MK-2206 include inhibition of Akt activation, induction of reactive oxygen species (ROS), and cross-talk between autophagy and apoptosis (Hirai et al., 2010; Cheng et al., 2011). In our previous studies, we showed that MK-2206 could enhance the efficacy of cisplatin and paclitaxel in both Akt-active SKOV3 and Akt-inactive ES2 ovarian cancer cells (Lin et al., 2015). Furthermore, MK-2206 in combination with a synthetic hemiasterlin derivative (*R*)(*S*)(*S*)-BF65 also exerted a synergistic cytotoxic effect in SKOV3 cells (Lai et al., 2016).

DNA double-strand breaks (DSBs) can lead to genome instability of cells. DSBs can occur endogenously or exogenously. Exogenously induced DSBs can be originated from some cancer treatment procedures, such as ionizing irradiation and chemotherapy. Failure of DSB repair will finally lead to cell death. Therefore, cells need to repair DSBs in order to preserve genome integrity. Homologous recombination (HR) and non-homologous end joining (NHEJ) are two major DSB repair systems (Chapman et al., 2012; Ceccaldi et al., 2016). The HR pathway is an error-free repair system functioning predominantly in S/G2 phases of the cell cycle when sister chromatids are available. NHEJ is an error-prone repair mechanism that can occur throughout the cell cycle and repair DSBs through blunt-end ligation. The recombinase Rad51 is recruited to DNA damage sites for DSB repair *via* HR. NHEJ is initiated by binding of the Ku70-Ku80 heterodimer to double-stranded DNA ends and DNA-dependent protein kinase is then recruited and activated to promote NHEJ. Chk2 serves as a checkpoint regulator leading to cell cycle arrest upon DNA damage. Chk1 activation can also initiate the DNA damage checkpoint response (Goodarzi and Jeggo, 2013).

ROS, such as H_2_O_2_ or superoxide, are produced mainly in mitochondria as by-products of cellular aerobic metabolism. Some anticancer agents, such as MK-2206, can also induce ROS generation (Cheng et al., 2011; Lin et al., 2015). ROS intermediates may produce oxidative damage to DNA, proteins, and lipids. Large amount of intracellular ROS can induce cell cycle arrest, senescence, and apoptosis (Liou and Storz, 2010). There are two main apoptotic pathways, the extrinsic and intrinsic pathways (Elmore, 2007). In response to extrinsic death receptor-mediated signals or intrinsic death signals, a series of caspases are activated by cleavage, leading to apoptosis. Poly(ADP-ribose) polymerase (PARP) is cleaved by caspases during apoptosis, and the ∼85-kDa cleaved PARP is usually used as a marker for apoptosis.

In search of anticancer drugs whose activity could be enhanced by WZB117, we found that the combination of MK-2206 and WZB117 showed the best synergistic cytotoxic effect against breast cancer cells, and further investigation revealed that MK-2206 and WZB117 exerted cytotoxic effect through inhibition of Akt, induction of ROS and DNA damage, as well as impairment of DNA damage repair.

## Materials and Methods

### Chemicals

MK-2206 (purity ≥98% by HPLC) was purchased from BioVision, (Mountain View, CA). Cisplatin, doxorubicin, WZB117 (purity ≥98% by HPLC), 2′,7′-dichlorodihydrofluorescein diacetate (DCFH-DA), and crystal violet were purchased from Sigma-Aldrich (St. Louis, MO). 3-(4,5-Dimethylthiazol-2-yl)-2,5-diphenyltetrazolium bromide (MTT), propidium iodide (PI), and 2-NBDG were obtained from Invitrogen Life Technologies (Carlsbad, CA). Stock solutions of MK-2206, doxorubicin, WZB117, and DCFH-DA were prepared in dimethyl sulfoxide (DMSO). Cisplatin and MTT were dissolved in phosphate-buffered saline (PBS). 2-NBDG was dissolved in water and crystal violet was dissolved in 20% methanol.

### Cell Culture, Drug Treatment, and Cell Viability Assays

Human breast cancer cell lines MCF-7 (originally from Michigan Cancer Foundation) and MDA-MB-231 (ATCC HTB-26, p32 from ATCC) were cultured in high-glucose Dulbecco’s modified Eagle’s medium supplemented with 10% FBS, 2 mM L-glutamine, and antibiotics (100 U/ml penicillin, 100 µg/ml streptomycin, and 0.25 µg/ml amphotericin B) at 37°C in a humidified 5% CO_2_ atmosphere. For viability assays, cells were seeded in 96-well plates (3–4 × 10^3^ cells/well) and subjected to drug treatments for indicated time periods followed by the MTT assay as described previously (Lin et al., 2015). Combination index (CI), an indicator of drug interactions in combination chemotherapy, was analyzed by CompuSyn software. Fraction of growth inhibition calculated from the MTT data and each corresponding concentration were entered to CompuSyn. The CI values at ED_50_ (CI_50_) were then calculated by CompuSyn software. CI values <1, = 1, and >1 refer to synergistic, additive, and antagonistic effects, respectively (Chou, 2010).

### Colony Formation Assay

Cells were seeded into a six-well plate (1,000 cells/well) and treated with drugs for 24 h. Then, drugs were removed and cells were allowed to grow in drug-free culture medium for 7–9 days. Colonies were rinsed with PBS, stained with 0.25% crystal violet, and photographed. Colonies with at least 50 cells were counted.

### 2-NBDG Flow Cytometric Analysis for Measuring Glucose Uptake

Cells were plated into 12-well plates, cultured overnight, rinsed with PBS, and then incubated with 200 µM of a fluorescent glucose derivative 2-NBDG (Yamada et al., 2007) in the presence of the vehicle control (DMSO) or indicated drugs in 500 µl of PBS for 1.5 h at 37°C in a CO_2_ incubator. Cells were harvested by trypsinization, resuspended in 500 µl of ice-cold PBS and then subjected to flow cytometric analysis by FACSCalibur (BD Biosciences, San Jose, CA). Geometric mean of fluorescence intensity of each sample was determined and the relative 2-NBDG uptake was calculated using the geometric mean of cells treated with the vehicle control as 100%.

### siRNA Transfection and Cell Viability Assays

MCF-7 cells were seeded into 96-well plates (5 × 10^3^ cells/well) and transfected with siRNAs using the Lipofectamine 2000 reagent. GLUT1 siRNA (SMARTpool) was obtained from Dharmacon and negative control siRNA was purchased from Santa Cruz (sc-37007). One day after transfection, cells were treated with drugs at indicated concentrations for 72 h and cell viability was measured by the MTT assay.

### Annexin V-FITC/PI Two-Dimensional Flow Cytometric Analysis

Cells were plated into six-wells and treated with indicated drugs for 48 h, then harvested for Annexin V-fluorescein isothiocyanate (FITC)/PI staining using an Annexin V apoptosis detection kit (Santa Cruz Biotechnology, Santa Cruz, CA) and subjected to two-dimensional flow cytometric analysis using FACSCalibur. At least 10,000 cells were analyzed for each sample using FlowJo software (Treestar, Ashland, OR).

### SDS-PAGE and Western Blot Analysis

After the indicated treatment, cells were harvested, washed with PBS, and lysed with ice-cold lysis buffer containing 10 mM Tris-HCl pH 7.5, 150 mM NaCl, 1% Triton X-100, 1 mM EDTA, 1 mM EGTA, 50 mM NaF, 1 mM Na_3_VO_4_, and protease inhibitor cocktail (Roche Diagnostics, Indianapolis, IN). Cleared cell lysates containing 20 µg of protein were denatured in sodium dodecyl sulfate (SDS) sample buffer, and then subjected to 10% SDS-PAGE and transferred to PVDF membrane. Western blot analysis was performed as previously described (Hsu and White, 1998) and image detection and quantification were performed using the ChemiDoc XRS system and Image Lab software (Bio-Rad Laboratories, Hercules, CA). Primary antibodies used were phospho-Akt (p-Akt, S473), total Akt, p-mTOR (S2448), total mTOR, p-p70S6K (T389), p70S6K, p-4E-BP1 (T37/46), 4E-BP-1, p-Chk1 (S345), p-Chk2 (T68), Ku80, γ-H2AX (S139) (Cell Signaling Technology, Boston, MA), p70S6K (Abcam PLC, Inc., Cambridge, MA), Rad51, Chk1, Chk2 (Santa Cruz Biotechnology), Bcl-2 (Dako, Carpinteria, CA), PARP (BD Biosciences), Caspase-3 (Imgenex Corp., San Diego, CA), GAPDH (Epitomics, San Diego, CA), and γ-tubulin (Sigma-Aldrich, St. Louis, MO). Secondary antibodies used were HRP-conjugated anti-mouse and anti-rabbit IgGs (Cell Signaling Technology). GAPDH was used as a loading control to normalize protein expression levels and relative protein levels were further calculated setting the levels of vehicle controls at 24 h as 1.

### Comet Assay

After drug treatment, cells were harvested by trypsinization, resuspended in ice-cold PBS, and 50 µl of cells was mixed with 100 µl of 1.5% prewarmed low melting point agarose in PBS. This mixture was loaded onto a frosted slide precoated with 0.7% agarose, and a coverslip was then applied to the slide. Slides were then submerged in pre-chilled lysis solution (2.5 M NaCl, 10 mM Tirs-base, 100 mM EDTA, and 1% Triton X-100 in PBS, pH 10.5) in the dark for 30 min at 4°C. After soaked with pre-chilled unwinding and electrophoresis buffer (0.03 N NaOH and 2 mM EDTA) for 30 min, slides were subjected to electrophoresis for 10–15 min at 0.5 V/cm (20 mA), stained with SYBR Green Gold, and comet images were visualized and captured using a fluorescence microscope (Carl Zeiss GmbH, Jena, Germany) with a ×20 objective. One hundred cells were counted to calculate the percentage of comet tail-positive cells.

### Immunofluorescence Staining

Cells were seeded into eight-well chamber slides (2–4 × 10^4^ cells/well). After overnight incubation, cells were treated with indicated drugs and subjected to immunofluorescence staining as described previously (Yu et al., 2008; Chen et al., 2014). Primary antibody used was Rad51 antibody (1:100 dilution, Santa Cruz) with FITC-conjugated anti-rabbit at a dilution of 1:200 as the secondary antibody or γ-H2AX (1:1,000 dilution) (Millipore, Billerica, MA) with Texas Red anti-mouse at a dilution of 1:200 as the secondary antibody. Nuclear counterstaining was performed using 4′,6-diamidino-2-phenylindole (DAPI), and slides were mounted with antifade (Invitrogen, Carlsbad, CA). Images were acquired on a fluorescence microscope (Carl Zeiss GmbH, Jena, Germany) with a ×100 objective. One hundred cells were scored for each sample, and the percentage of cells with at least five Rad51 nuclear foci (defined as Rad51-positive cells) or stained positive for γ-H2AX was calculated.

### HR Assay

Cells were seeded into a six-well plate (3 × 10^5^ cells/well) and transfected with 0.8 µg pDR-GFP and 0.8 µg pCMV-I-*Sce*I (Pierce et al., 1999) using Lipofectamine 2000 (Invitrogen, Carlsbad, CA). One day after transfection, cells were treated with DMSO or indicated drugs for 24 h, harvested by trypsinization, and resuspended in ice-cold 1× PBS and then subjected to flow cytometric analysis using FACSCalibur on a two-dimensional dot plot of the GFP fluorescence (FL1) and cell autofluorescence (FL2). At least 25,000 cells were analyzed by FlowJo software.

### NHEJ Assay

Cells were seeded into a six-well plate (3 × 10^5^ cells/well) and transfected with 300 ng of pGL3-Control plasmid linearized by *Hin*dIII digestion (Promega, Madison, WI). One day after transfection, cells were treated with DMSO or indicated drugs for 24 h, and then subjected to the luciferase assay using a Luciferase Assay System (Promega, Madison, WI) according to the manufacturer’s instructions. Briefly, cells were rinsed with 1× PBS and then incubated with lysis reagent for 15 min on ice. Cells were scraped and the lysate was transferred to a microfuge tube. Cleared lysate after brief centrifugation to remove cell debris was transferred to a white 96-well microtiter plate and mixed with Luciferase Assay Reagent. Luminescence was measured by Orion II Microplate Luminometer (Berthold Technologies, Bad Wildbad, Germany) and normalized with the protein concentration to quantify the NHEJ efficiency. The *Hin*dIII restriction enzyme cuts the pGL3-Control plasmid between the SV40 promoter and the *luc*+ coding sequence. Luminescence can only be detected when the linearized plasmid is re-ligated by NHEJ and luciferase is expressed inside the cell. Luminescence in lysate of pGL3-Basic (with *luc*+ coding sequence but without an upstream promoter to drive luciferase expression) transfected cells was measured and served as the background signal.

### Detection of Intracellular ROS Generation

Cells were incubated with DMSO (vehicle control), MK-2206, WZB117, or the combination of MK-2206 and WZB117 along with 10 µM DCFH-DA for 30 min at 37°C, harvested by trypsinization, resuspended in ice-cold 1× PBS, and then subjected to flow cytometric analysis. Cells with ROS production were quantified.

### Statistical Analysis

Data are presented as the mean ± SEM of at least three independent experiments. Statistical analysis of data for multiple groups was performed using one-way ANOVA followed by the Bonferroni *t* test (GraphPad Prism 6, GraphPad Software Inc., La Jolla, CA). For comparison of two groups, statistical significance was assessed using the two-sided Student’s *t* test. *P* values less than 0.05 were considered statistically significant (*P < 0.05, **P < 0.01, ***P < 0.001).

## Results

### Synergistic Growth Inhibitory Effect of MK-2206 and WZB117 in MCF-7 and MDA-MB-231 Cells

In a preliminary test, anticancer drugs including cisplatin, doxorubicin, and MK-2206 were combined with WZB117, and the allosteric Akt inhibitor MK-2206 showed the best synergistic anticancer effect with WZB117 in breast cancer cells (**Supplementary Figure S1**). The combination of MK-2206 and WZB117 was then characterized in breast cancer cell lines including ER(+) MCF-7 and triple-negative MDA-MB-231 cells.

**Figure 1:**
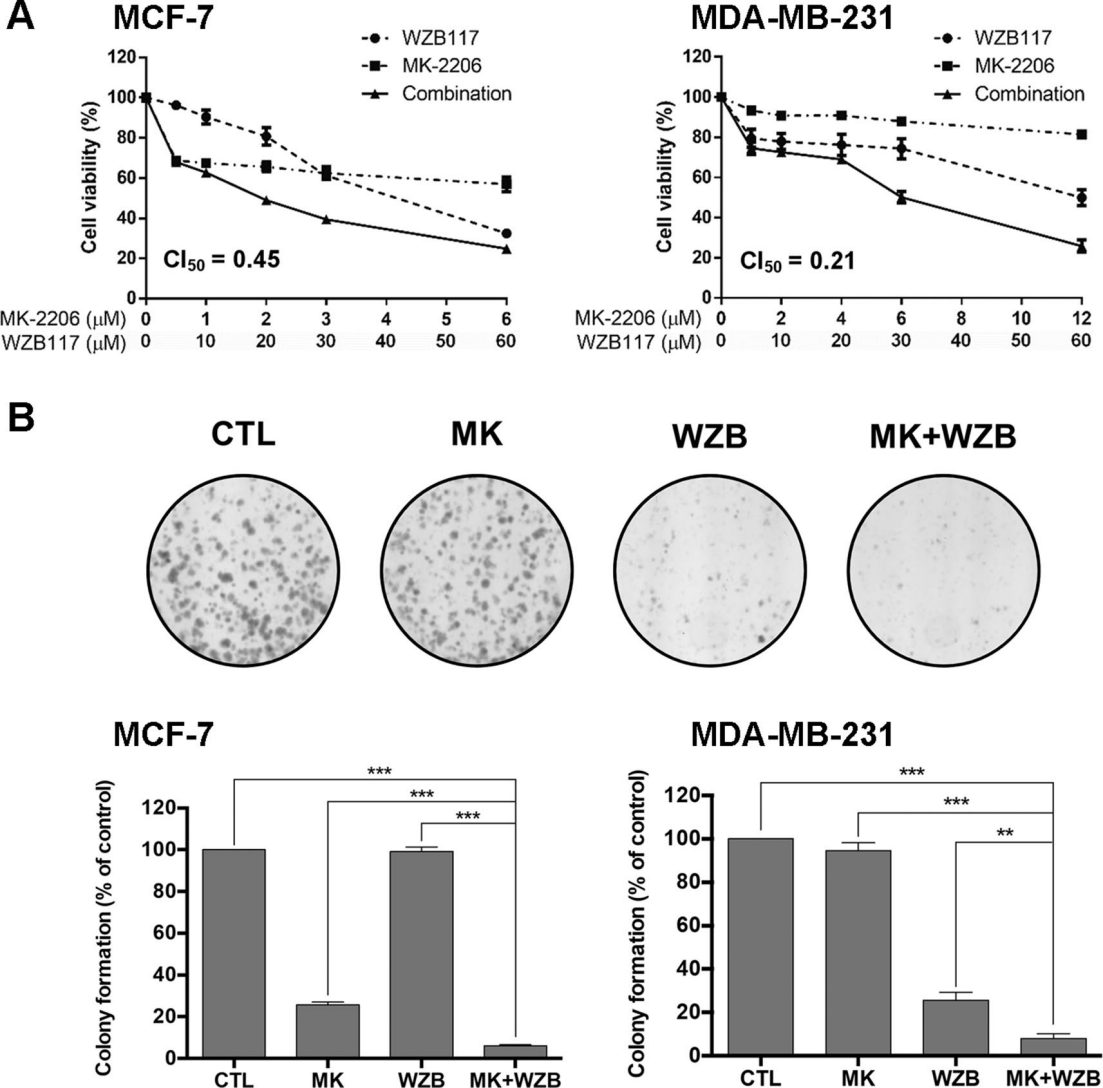
MK-2206 and WZB117 synergistically inhibit the growth of MCF-7 and MDA-MB-231 breast cancer cells. **(A)** Dose–response curves of cells treated with MK-2206 and WZB117 either alone or in combination for 48 h. Cell viability was measured by the MTT assay. **(B)** MK-2206 and WZB117 inhibit colony formation. A set of colony formation images of MDA-MB-231 cells are illustrated in the *upper panels* and quantitative results of colony formation are shown in the *lower panels*. MCF-7 cells were treated with 0.5 µM MK-2206 and/or 5 µM WZB117, and MDA-MB-231 cells were treated with 6 µM MK-2206 and/or 30 µM WZB117 for 24 h, then cultured in drug-free culture medium for 7 days (MCF-7) or 9 days (MDA-MB-231), and colonies were then scored. *CTL*, the DMSO vehicle control; *MK*, MK-2206; *WZB*, WZB117, *MK+WZB*, the combination of MK-2206 and WZB117. Data are presented as the mean ± SEM of three independent experiments. ** P < 0.01; *** P < 0.001.

The synergistic effect of drug combination is dependent on the molar ratio. Preliminary tests were conducted based on the potency of the drugs, e.g., the ratio of IC_50_ of both drugs, to obtain an optimal molar ratio (Chou, 2006). MCF-7 cells were treated for 48 h with MK-2206 (0–6 µM) and WZB117 (0–60 µM) alone or in combination with a molar ratio of 1:10 based on results from preliminary tests. Cell viability was measured by the MTT assay. Combination index (CI), an indicator of drug interactions, was calculated based on the results of MTT assays using CompuSyn software. CI value at 50% growth inhibition (CI_50_) of the MK-2206 and WZB117 combination was 0.45, indicating a strong synergistic growth inhibition in MCF-7 cells (**Figure 1A**, left panel). MDA-MB-231 cells were less sensitive to MK-2206. However, a strong synergistic effect was also observed in MDA-MB-231, with a CI_50_ of 0.21 at a molar ratio of 1:5 (MK-2206 vs. WZB117) (**Figure 1A**, right panel).

The long-term growth inhibitory effect was examined by colony formation assay. MCF-7 cells were treated with the DMSO vehicle control, 0.5 µM MK-2206, 5 µM WZB117, or the combination of MK-2206 and WZB117 for 24 h and then incubated with drug-free medium for 7 days. MDA-MB-231 cells were treated with the DMSO control, 6 µM MK-2206, 30 µM WZB117, or the combination for 24 h and then incubated with drug-free medium for 9 days. A set of colony formation results from MDA-MB-231 cells is shown in **Figure 1B**, upper panels, and quantitative data of three independent experiments from both cell lines are shown in **Figure 1B**, lower panels. The results indicated that the combination of MK-2206 and WZB117 effectively suppressed colony formation and was significantly more efficient than single agents. Nevertheless, higher concentrations of both drugs were required to suppress colony formation in MDA-MB-231 triple-negative breast cancer cells.

**Figure 2:**
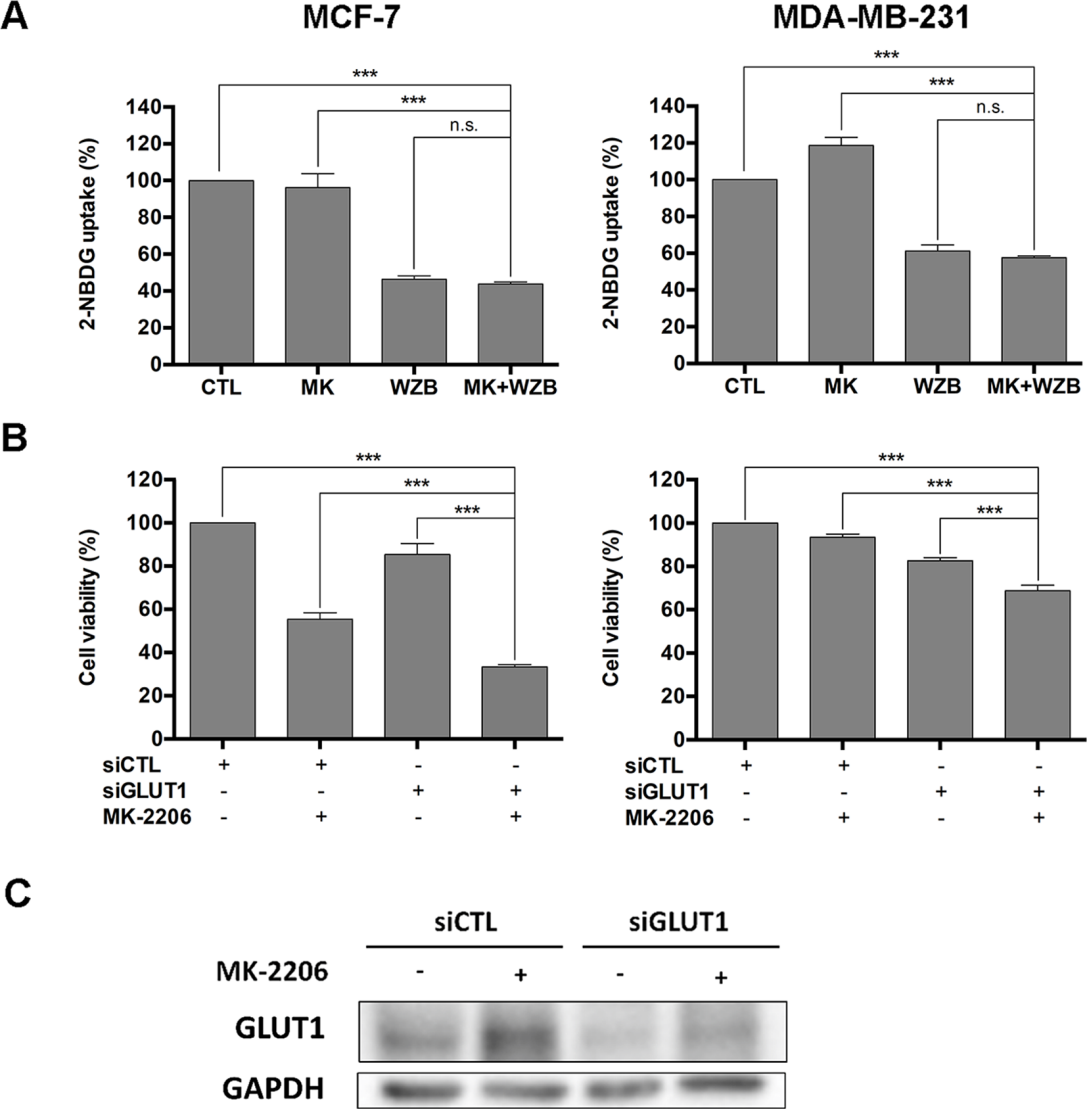
WZB117 may enhance the growth inhibitory effect of MK-2206 *via* inhibition of GLUT1 in MCF-7 and MDA-MB-231 cells. **(A)** WZB117 inhibits glucose uptake in MCF-7 and MDA-MB-231 cells. Cells were incubated with 2-NBDG in the presence of DMSO, 6 µM MK-2206 and/or 60 µM WZB117 (MCF-7) or 12 µM MK-2206 and/or 60 µM WZB117 (MDA-MB-231) for 1.5 h and harvested for flow cytometric analysis of 2-NBDG fluorescence, and relative 2-NBDG uptake was calculated. Data are presented as the mean ± SEM of three independent experiments. **(B)** Knockdown of GLUT1 enhances the growth inhibitory effect of MK-2206 in MCF-7 and MDA-MB-231 cells. Cells were transfected with control siRNA (siCTL) or GLUT1 siRNA (siGLUT1) and then treated with MK-2206 (6 µM in MCF-7 cells or 12 µM in MDA-MB-231 cells) for 72 h, followed by the MTT assay to assess cell viability. Data are presented as the mean ± SEM of three independent experiments. **(C)** Western blot analysis of GLUT1 in MCF-7 cells after siRNA transfection and MK-2206 treatment. MCF-7 cells were transfected with control siRNA (siCTL) or GLUT1 siRNA (siGLUT1), allowed to recover for 48 h, and then treated with 6 µM MK-2206 for 48 h before harvested for Western blot analysis. *** P < 0.001, n.s., not significant (P > 0.05).

WZB117 was previously reported as a potent GLUT1 inhibitor (Liu et al., 2012). Relative 2-NBDG uptake results obtained from flow cytometric analysis shown in **Figure 2A** indicated that 60 µM of WZB117 inhibited 53.6% and 38.9% of glucose uptake in MCF-7 and MDA-MB-231 cells, respectively. This effect was comparable to that on growth inhibition as illustrated in **Figure 1A** (67.6% and 50.1% in MCF-7 and MDA-MB-231 cells, respectively). The combination with MK-2206 only slightly enhanced the effect of WZB117, but the difference was not statistically significant.

To further verify that WZB117 acted *via* inhibition of GLUT1, MCF-7 and MDA-MB-231 cells were transfected with control siRNA (siCTL) or GLUT1 siRNA (siGLUT1) to knock down GLUT1 and then treated with vehicle control or MK-2206 (6 µM for MCF-7 cells and 12 µM for MDA-MB-231 cells) for 48 h, followed by the MTT assay to assess cell viability. As illustrated in **Figure 2B**, the combination of siGLUT1 and MK-2206 was significantly more effective than single treatments or untreated control in growth inhibition. These data suggested that inhibition of GLUT1 by WZB117 at least in part accounted for the synergistic effect of WZB117 and MK-2206. Interestingly, as illustrated in **Figure 2C**, MK-2206 induced an increase in the GLUT1 protein level, which was reversed by siGLUT1 transfection in MCF-7 cells. This may also partly explain why WZB117 can enhance the activity of MK-2206.

### The Combination of MK-2206 and WZB117 Induces Apoptosis

**Figure 3:**
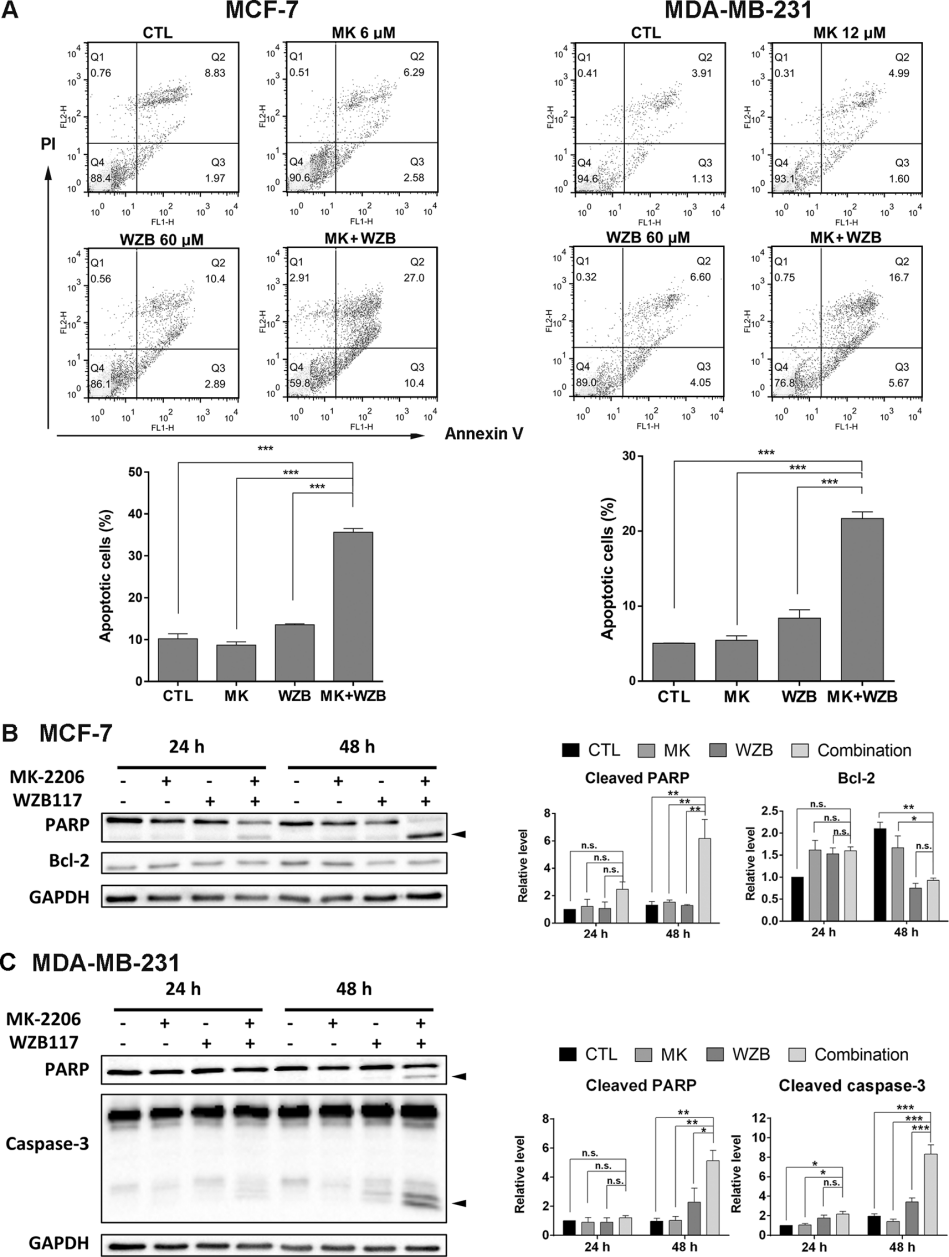
MK-2206 and WZB117 synergistically induce apoptosis in MCF-7 and MDA-MB-231 cells. **(A)** Annexin V-FITC/PI staining and flow cytometric analysis. Cells were treated with 6 or 12 µM of MK-2206 and 60 µM of WZB117 either alone or in combination for 48 h. Representative dot plots and quantitative data from three independent experiments showed that MK-2206 and WZB117 significantly induced apoptosis. **(B)** Western blot analysis of proteins involved in apoptosis in MCF-7 cells. Cells were treated with 6 µM of MK-2206 and 60 µM of WZB117 either alone or in combination for 24 or 48 h. **(C)** Western blot analysis of proteins involved in apoptosis in MDA-MB-231 cells. Cells were treated with 12 µM of MK-2206 and 60 µM of WZB117 either alone or in combination for 24 or 48 h. Quantitative data are presented as the mean ± SEM of three independent experiments. Cleaved PARP and caspase-3 are marked with *arrowheads*. * P < 0.05; ** P < 0.01; *** P < 0.001; n.s., not significant (P > 0.05).

Annexin V-FITC/PI double staining was performed to determine whether MK-2206 and WZB117 induced apoptosis in breast cancer cells. MCF-7 cells were treated with 6 µM MK-2206 and/or 60 µM WZB117 for 48 h and harvested for Annexin V-FITC/PI staining and flow cytometric analysis. Representative flow cytometric dot plots and quantitative results from three independent experiments are shown in **Figure 3A**, left panel. Apoptotic cells (early apoptosis and late apoptosis) in the MK-2206/WZB117 combination group (35.6%) were significantly increased compared to the vehicle control (10.2%), MK-2206 (8.65%), or WZB117 (13.5%). MDA-MB-231 cells treated with 12 µM MK-2206 and/or 60 µM WZB117 for 48 h also showed similar results, with 5.06%, 5.45%, 8.41%, and 21.7% of apoptotic cells in the control, MK-2206, WZB117, and combination groups, respectively (**Figure 3A**, right panel).

Western blot analysis was conducted next. For all the Western blot analyses, MCF-7 cells were treated with 6 µM MK-2206 and/or 60 µM WZB117, and MDA-MB-231 cells were treated with 12 µM MK-2206 and/or 60 µM WZB117 for 24 or 48 h. In MCF-7 cells, PARP cleavage was apparent at 24 h and increased significantly at 48 h only when cells were treated with the combination of MK-2206 and WZB117 (**Figure 3B**). Although anti-apoptotic protein Bcl-2 was moderately induced by MK-2206, WZB117, or the combination of both relative to the vehicle control at 24 h, it was significantly downregulated by either WZB117 alone or the MK-2206/WZB117 combination at 48 h, with *P* values of 0.0018 and 0.0016, respectively (two-sided *t* test), compared to the vehicle control (relative Bcl-2 levels: 48 h CTL, 2.10; WZB117, 0.75; combination, 0.93; 24 h vehicle control was set as 1) (**Figure 3B**). In MDA-MB-231 cells, PARP cleavage was clearly induced after treatment with the combination of MK-2206 and WZB117 for 48 h. Caspase-3 cleavage, which was undetectable in MCF-7 cells, was also significantly induced by the MK-2206/WZB117 combination in MDA-MB-231 cells at 48 h (**Figure 3C**).

Taken together, these data indicated that the combination of MK-2206 and WZB117 synergistically induced apoptosis in MCF-7 and MDA-MB-231 cells.

### The Effect of MK-2206 and WZB117 on Akt/mTOR Signaling in MCF-7 and MDA-MB-231 Cells

To determine whether the Akt/mTOR pathway was involved in the growth inhibitory effect of MK-2206 and WZB117, several proteins involved in Akt/mTOR signaling were analyzed by Western blot analysis.

**Figure 4:**
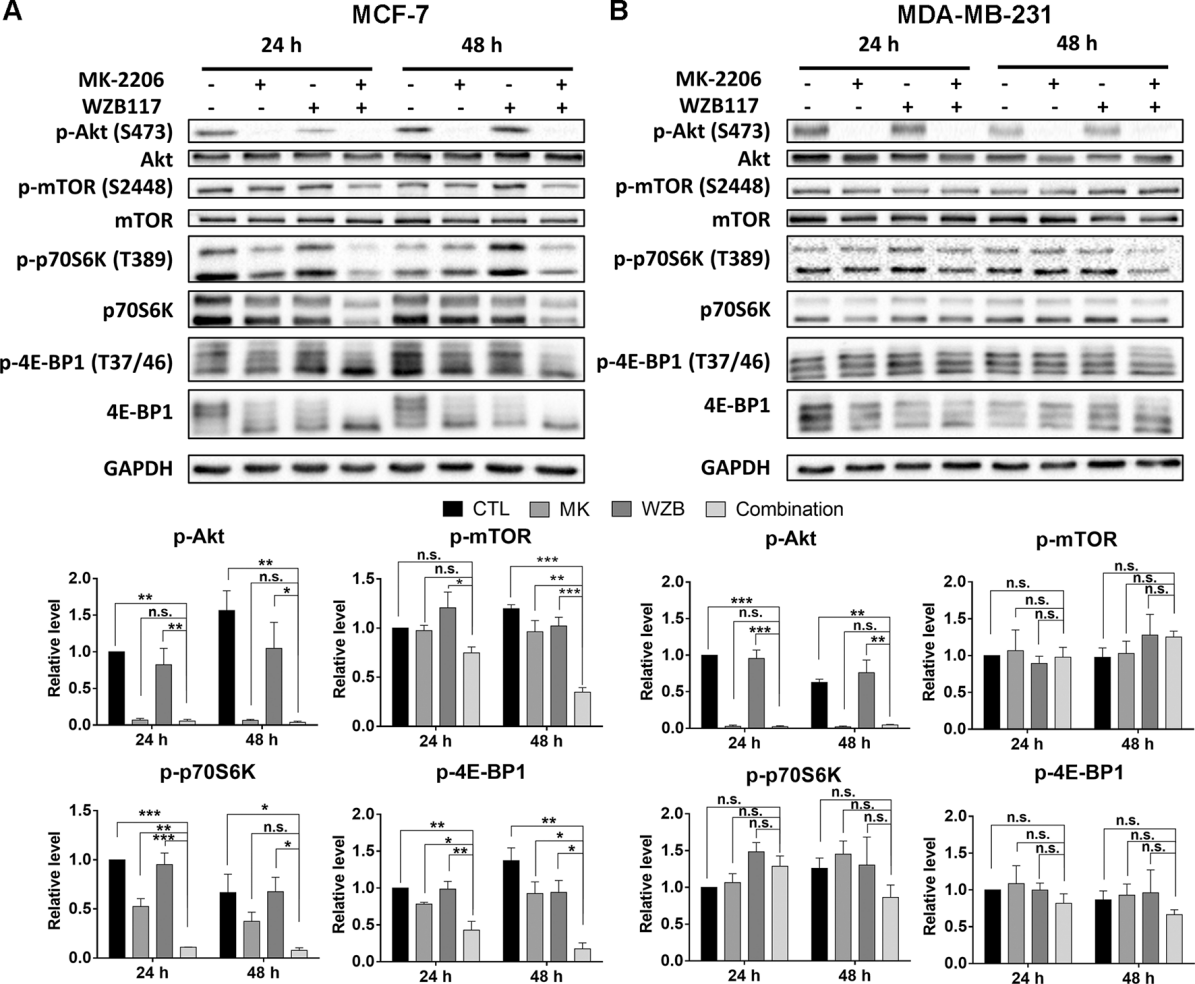
The effect of MK-2206 and WZB117 on proteins involved in the Akt/mTOR signaling pathway. **(A)** MCF-7 cells. **(B)** MDA-MB-231 cells. MCF-7 cells were treated with 6 µM MK-2206 and 60 µM WZB117, and MDA-MB-231 cells were treated with 12 µM MK-2206 and 60µM WZB117, either alone or in combination, for 24 or 48 h. Cells were then harvested for Western blot analysis. Quantitative data are presented as the mean ± SEM of three independent experiments. *P < 0.05; **P < 0.01; ***P < 0.001; n.s., not significant (P > 0.05).

In MCF-7 cells, MK-2206 and the MK-2206/WZB117 combination effectively blocked the phosphorylation of Akt. WZB117 also slightly downregulated p-Akt compared to the vehicle control, but the differences were not statistically significant. The combination of MK-2206 and WZB117 further downregulated some of the downstream effectors of Akt, including p-mTOR, p-p70S6K, and p-4E-BP1, compared to single agents alone. Intriguingly, total p70S6K and 4E-BP1 proteins were also downregulated by the combination treatment (**Figure 4A**).

In MDA-MB-231 cells, the combination of MK-2206 and WZB117 also dramatically inhibited Akt. However, the effect on p-mTOR, p-p70S6K, and p-4E-BP1 was not as obvious as in MCF-7 cells (**Figure 4B**), suggesting other Akt downstream targets may be involved in the growth inhibition of MK-2206 and WZB117-treated MDA-MB-231 cells.

### MK-2206 and WZB117 Induce DNA Damage in MCF-7 and MDA-MB-231 Cells

**Figure 5:**
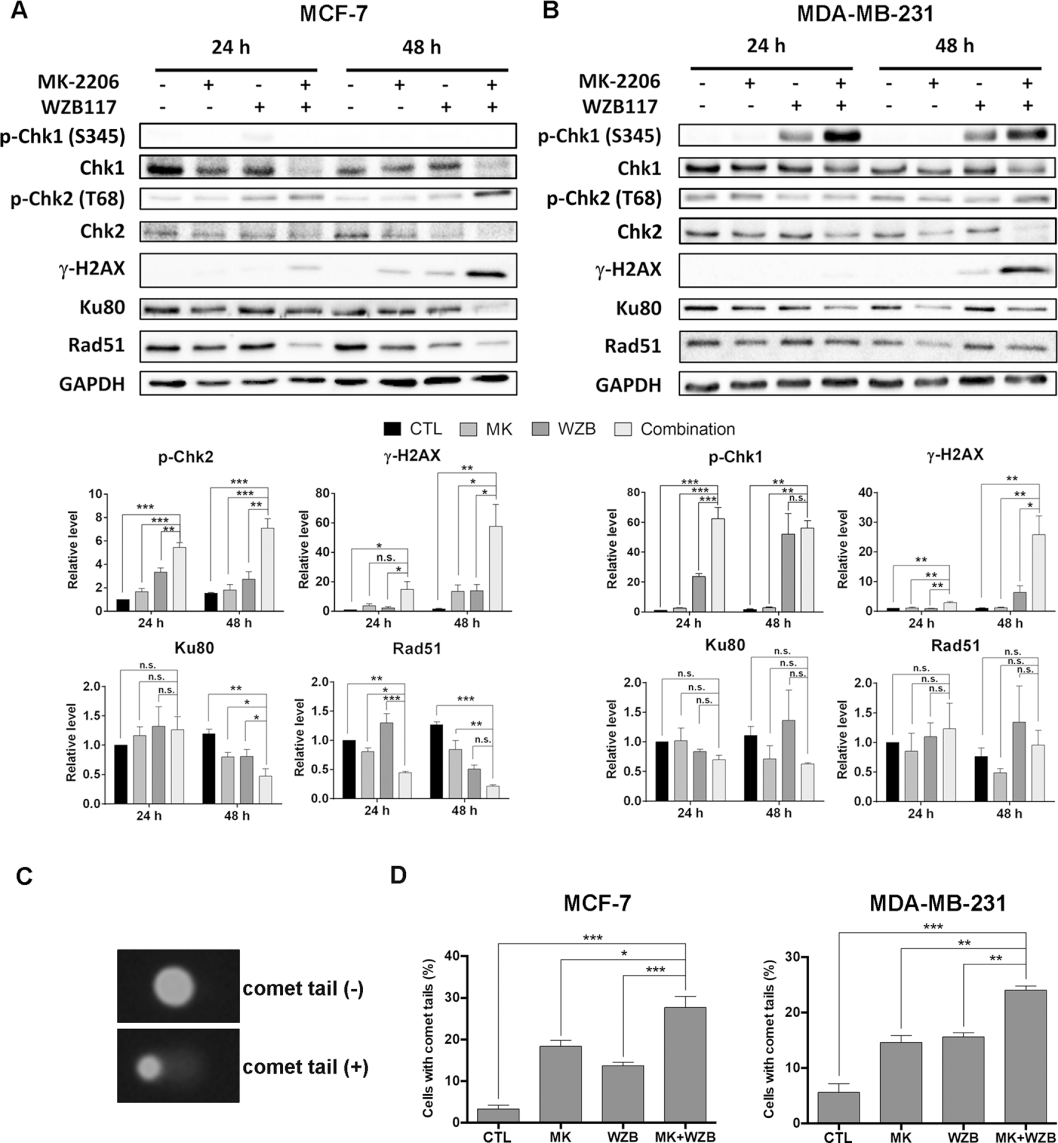
MK-2206 and WZB117 affect proteins involved in DNA damage signaling and repair, determined by Western blot analysis, and induce DNA damage, determined by the comet assay. **(A)** MCF-7 cells. **(B)** MDA-MB-231 cells. MCF-7 cells were treated with 6 µM MK-2206 and 60 µM WZB117 and MDA-MB-231 cells were treated with 12 µM MK-2206 and 60 µM WZB117, either alone or in combination, for 24 or 48 h. Cells were then harvested for Western blot analysis. Quantitative data are presented as the mean ± SEM of three independent experiments. **(C)** Comet images. **(D)** Quantitative data of comet assay. MCF-7 cells were treated with 6 µM MK-2206 and/or 60 µM WZB117 for 1 h and MDA-MB-231 cells were treated with 12 µM MK-2206 and/or 60 µM WZB117 for 6 h and subjected to alkaline comet assay. Data are presented as the mean ± SEM of three independent experiments. *P < 0.05; **P < 0.01; ***P < 0.001; n.s., not significant (P > 0.05).

MK-2206 in combination with a hemiasterlin derivative (*R*)(*R*)(*S*)-BF65 was previously reported to induce γ-H2AX, an indicator of DNA damage, in ovarian cancer cells (Lai et al., 2016). We then determined whether the combination of MK-2206 and WZB117 could also cause DNA damage. As illustrated in **Figure 5A**, Chk2 phosphorylation at threonine 68 (p-Chk2) and γ-H2AX were induced in MCF-7 cells treated with MK-2206 and WZB117 either alone or in combination, and the combination treatment significantly increased the level of p-Chk2 and γ-H2AX in a time-dependent manner. In MDA-MB-231 cells, Chk1 phosphorylation at serine 345 (p-Chk1) instead of p-Chk2 was induced by WZB117 and more dramatically by the MK-2206/WZB117 combination at both 24 and 48 h. γ-H2AX was also markedly induced by the combination of MK-2206 and WZB117 at 48 h (**Figure 5B**). These data revealed that MK-2206 or WZB117 only slightly induced DNA damage; however, combination treatment significantly enhanced the DNA damaging effect in both MCF-7 and MDA-MB-231 cells. Interestingly, DNA damage response was mediated through Chk2 in MCF-7 cells, but *via* Chk1 in MDA-MB-231 cells.

To examine DNA damaging effect upon shorter drug exposure, alkaline comet assay was conducted to measure DNA integrity after drug treatment. Representative MCF-7 cells with or without comet tails are shown in **Figure 5C** and quantitative results from three independent experiments of MCF-7 cells treated with 6 µM MK-2206 and/or 60 µM WZB117 for 1 h are shown in **Figure 5D**, left panel. Quantitative results of MDA-MB-231 cells treated with 12 µM MK-2206 and/or 60 µM WZB117 for 6 h are shown in **Figure 5D**, right panel. The results showed that even a short time period of treatment with MK-2206 and WZB117 caused DNA damage and the percentage of cells with comet tails in combination treatment was significantly higher than single agents alone (**Figure 5D**), indicating that DNA damage induced by MK-2206 and WZB117 could be an early event which may ultimately lead to apoptosis.

### MK-2206 and WZB117 May Compromise DNA Repair in MCF-7 and MDA-MB-231 Cells

Proteins participating in DNA repair were also evaluated by Western blot analysis. Ku80, an important DNA sensor in the NHEJ system, was significantly downregulated after 48 h of combination treatment in MCF-7 cells compared to the vehicle control or single treatments (**Figure 5A**). Ku80 protein level was also decreased after combination treatment in MDA-MB-231, but the result did not reach statistical significance (**Figure 5B**). Furthermore, Rad51, which plays a major role in HR repair, was significantly downregulated by combination treatment for 24 and 48 h in MCF-7 cells (0.44- and 0.17-fold of the respective controls) (**Figure 5A**), but was not significantly affected in MDA-MB-231 cells (**Figure 5B**).

**Figure 6:**
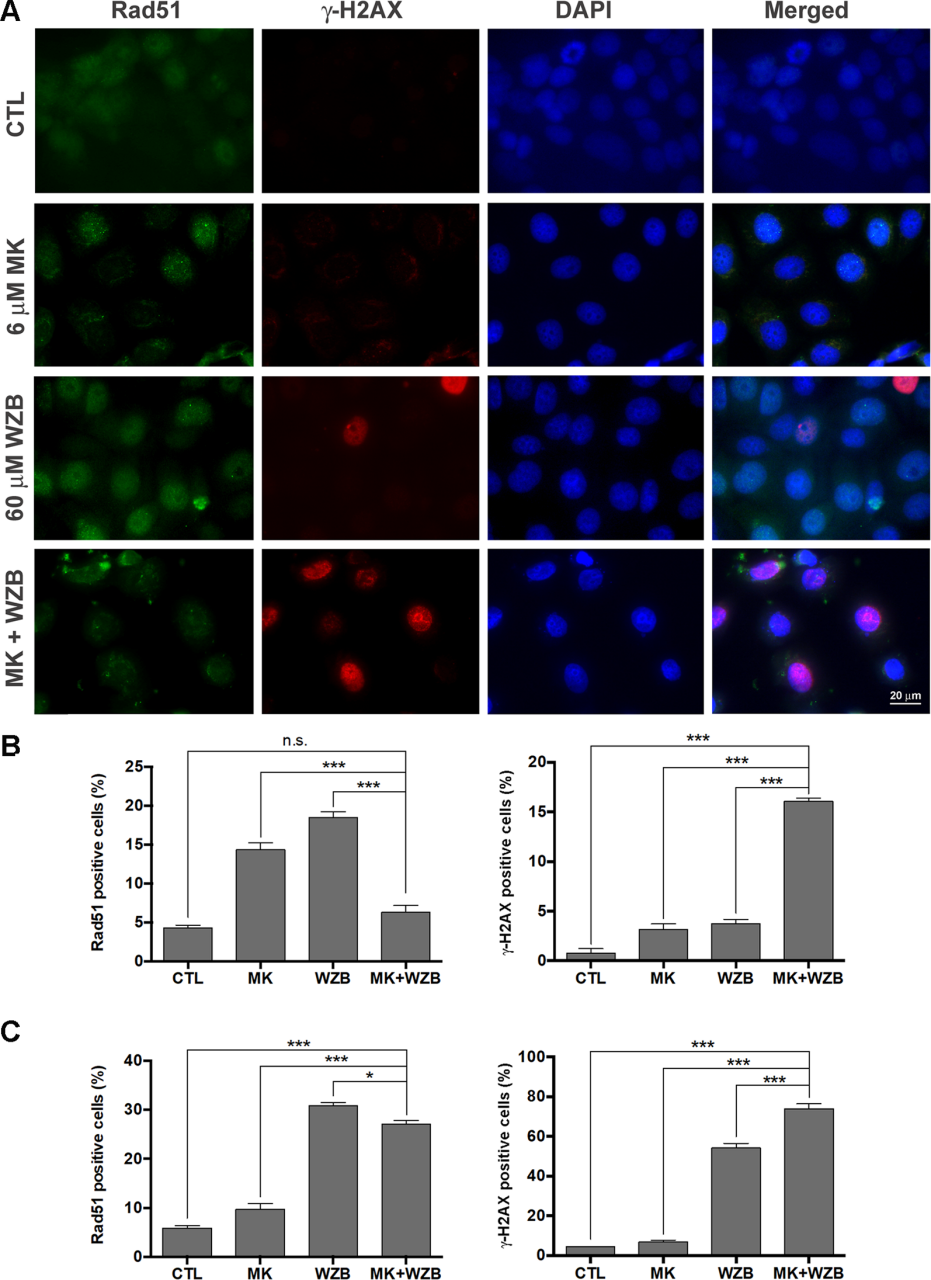
MK-2206 and WZB117 induce DNA damage and may compromise DNA repair. **(A)** Images of MCF-7 cells stained with Rad51 (*green*) and γ-H2AX (*red*) 24 h after drug treatment. The nucleus was counterstained with DAPI (*blue*). *Scale bar*, 20 µm. **(B)** Quantitative data of Rad51-positive and γ-H2AX-positive MCF-7 cells. **(C)** Quantitative data of Rad51-positive and γ-H2AX-positive MDA-MB-231 cells. MCF-7 cells were treated with 6 µM MK-2206 and/or 60 µM WZB117 and MDA-MB-231 cells were treated with 12 µM MK-2206 and/or 60 µM WZB117 for 24 h. Rad51-positive cells were defined as those with five or more Rad51 nuclear foci. Data are presented as the mean ± SEM of three independent experiments. * P < 0.05; *** P < 0.001; n.s., not significant (P > 0.05).

Rad51 foci in the nucleus, representing ongoing HR repair, and γ-H2AX staining, indicating DNA damage, were then evaluated after 24 h of drug treatment. Representative results of MCF-7 cells treated with 6 µM MK-2206 and/or 60 µM WZB117 are shown in **Figure 6A** and quantitative results from three independent experiments are shown in **Figure 6B**. Consistent with the Western blot results shown in **Figure 5A**, γ-H2AX(+) cells were dramatically increased after combination treatment in MCF-7 cells (16.1% vs. 0.78%, 3.14%, and 3.76% in the control, MK-2206, and WZB117 groups, respectively) (**Figure 6B**, right panel). Rad51 foci were significantly increased with 14.4% and 18.5% of Rad51(+) cells after MK-2206 and WZB117 single treatments, respectively, compared to 4.32% in the control. In contrast, Rad51 foci formation was significantly inhibited in the combination group, with only 6.3% of Rad51(+) cells (**Figure 6B**, left panel). Quantitative results of MDA-MB-231 cells treated with 12 µM MK-2206 and/or 60 µM WZB117 are shown in **Figure 6C**. The combination group induced significantly more γ-H2AX(+) cells (73.9%) compared to the other groups (4.40%, 6.96%, and 54.2% in the control, MK-2206, and WZB117 groups, respectively) and less Rad51(+) cells (27.11%) compared to WZB117 alone (30.8%). Interestingly, MK-2206 alone did not increase much, but WZB117 alone induced more γ-H2AX(+) or Rad51(+) cells in MDA-MB-231 cells. Although the combination of MK-2206 and WZB117 did not suppress the formation of Rad51 foci as obviously as in MCF-7 cells, it caused the accumulation of much more γ-H2AX(+) cells, suggesting that HR mechanism was initiated to some extent but DNA damage repair was still defective in MDA-MB-231 cells.

**Figure 7:**
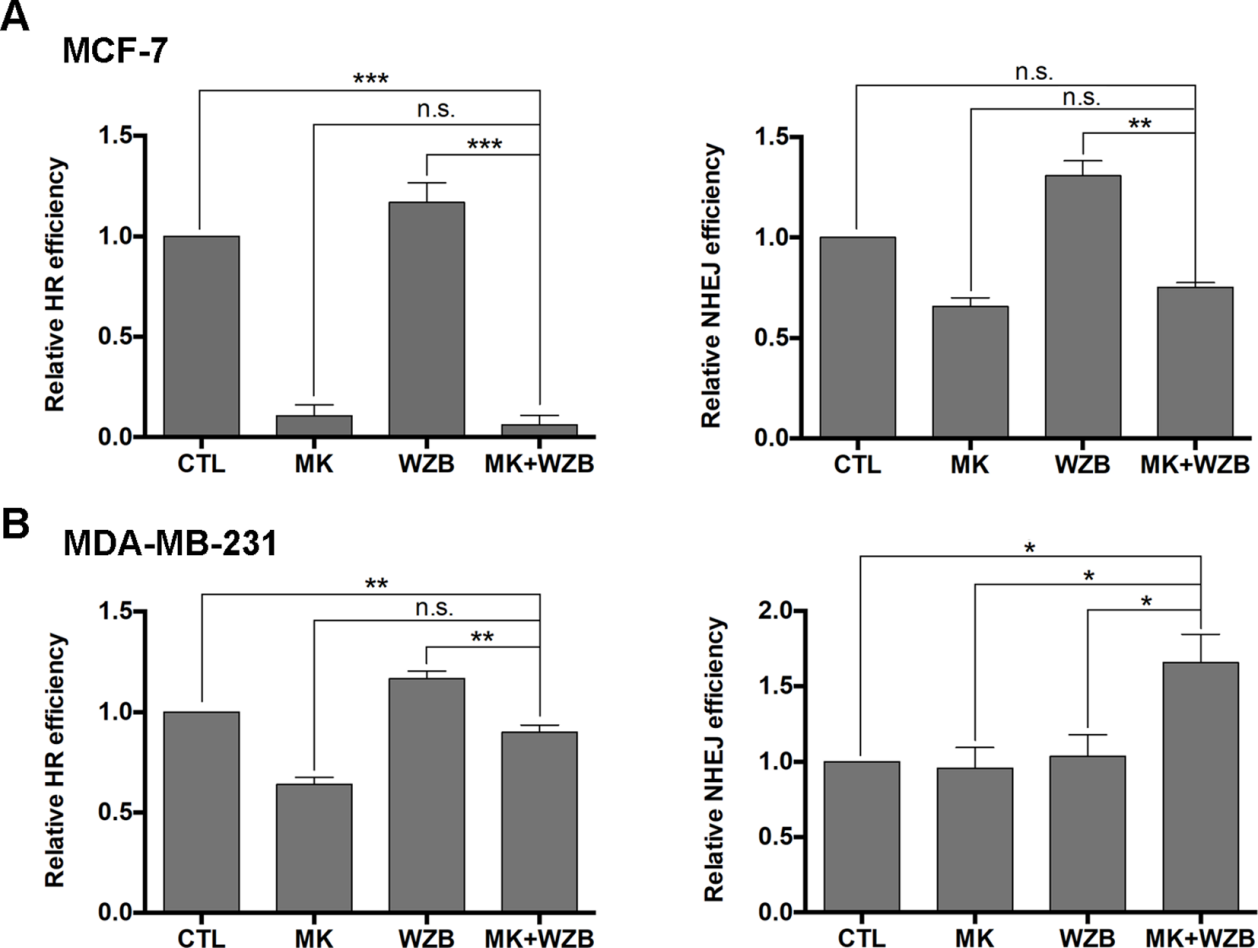
The MK-2206 and WZB117 combination affects DNA repair *via* HR and NHEJ. **(A)** HR and NHEJ assays in MCF-7 cells. **(B)** HR and NHEJ assays in MDA-MB-231 cells. For drug treatment, MCF-7 cells were treated with 6 µM MK-2206 and/or 60 µM WZB117 and MDA-MB-231 cells were treated with 12 µM MK-2206 and/or 60 µM WZB117 for 24 h. Data are presented as the mean ± SEM of three independent experiments, except NHEJ assay in MCF-7 cells (*N* = 2).*P < 0.05; **P < 0.01; ***P < 0.001; n.s., not significant (P > 0.05).

Assays were also performed to measure relative HR and NHEJ frequencies. As shown in **Figure 7A**, HR and NHEJ were significantly downregulated by MK-2206 or the combination of MK-2206 and WZB117, but slightly upregulated by WZB117 in MCF-7 cells. In MDA-MB-231 cells, HR was inhibited by MK-2206 and slightly upregulated by WZB117, and the MK-2206/WZB117 combination showed an intermediate effect. MK-2206 or WZB117 alone did not affect NHEJ in MDA-MB-231 cells, but the MK-2206/WZB117 combination enhanced NHEJ efficiency (**Figure 7B**).

Taken together, these results indicate that the combination of MK-2206 and WZB117 not only lead to DNA damage but may also affect DNA repair in MCF-7 and MDA-MB-231 cells.

### MK-2206 and WZB117 Significantly Induce ROS Within a Very Short Time

**Figure 8:**
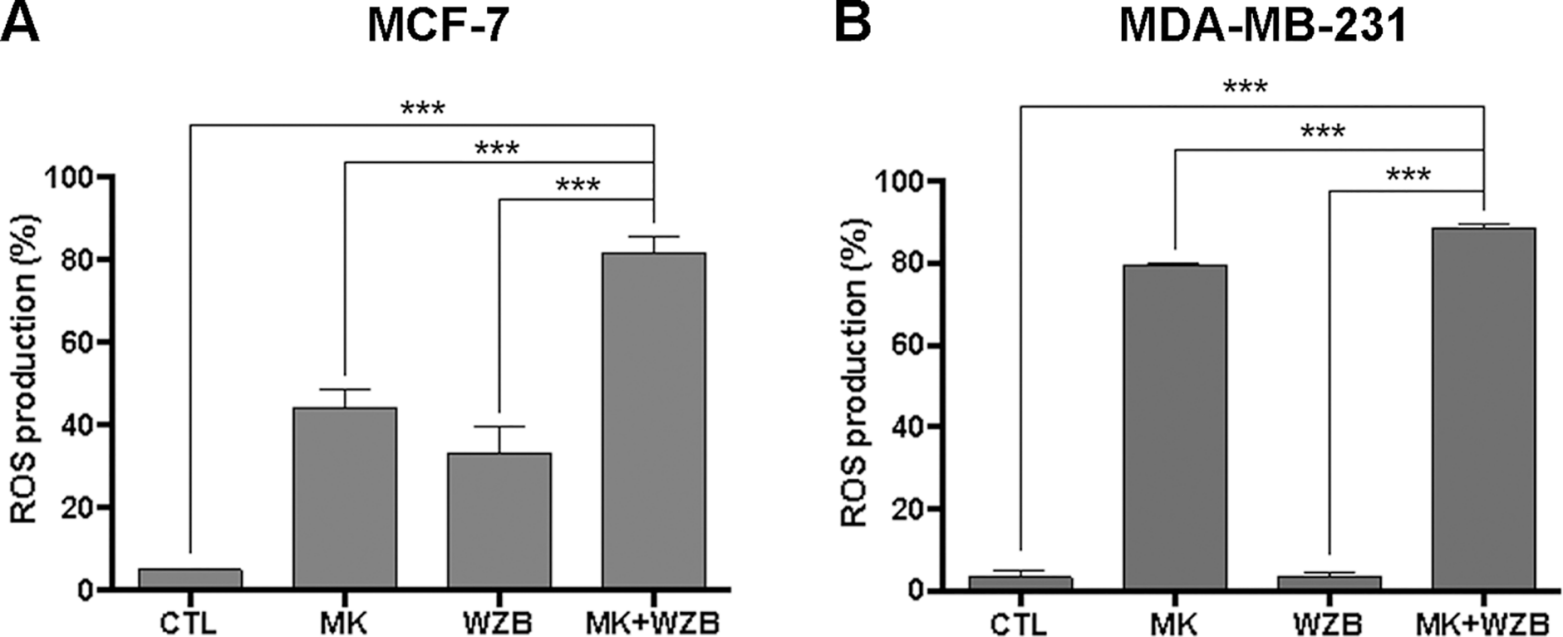
MK-2206 and WZB117 synergistically induce ROS in breast cancer cells. **(A)** Quantitative data of MCF-7 cells with ROS production. Cells were treated with 3 µM MK-2206 and 30 µM WZB117 either alone or in combination along with 10 µM DCFH-DA for 30 min. **(B)** Quantitative data of MDA-MB-231 cells with ROS production. Cells were treated with 6 µM MK-2206 and 30 µM WZB117 either alone or in combination along with 10 µM DCFH-DA for 30 min. DCFH-DA is non-fluorescent and is hydrolyzed by intracellular esterases to 2,7-dichlorodihydrofluorescein, which is then oxidized to fluorescent 2,7-dichlorofluorescein (DCF) by ROS. DCF fluorescence was detected by flow cytometric analysis and 10,000 cells were counted. The percentage of cells with ROS production was indicated. Data are presented as the mean ± SEM of at least three independent experiments. *** P < 0.001.

Previous studies have shown that increased levels of ROS can lead to DNA damage and apoptosis (Liou and Storz, 2010). MK-2206 has been reported to increase the intracellular ROS level (Lin et al., 2015; Chen et al., 2017b). Therefore, we next determined whether MK-2206 and WZB117 modulated ROS generation in MCF-7 and MDA-MB-231 cells. As illustrated in **Figure 8A**, 3 µM MK-2206 or 30 µM WZB117 clearly induced ROS within 30 min in MCF-7 cells (44.4% or 33.1% vs. 5.04% in the vehicle control), and the combination of MK-2206 and WZB117 further enhanced ROS production (81.7%). The results correlated well with those from the comet assay (**Figure 5D**, left panel) and γ-H2AX induction (**Figures 5A** and **6B**, right panel), suggesting that ROS production may play a role in DNA damage induction in MCF-7 cells.

In MDA-MB-231 cells, 6 µM MK-2206 but not 30 µM WZB117 dramatically induced ROS production (79.6% and 3.5%, respectively, vs. 3.34% in the vehicle control), and the combination of both further enhanced ROS production (88.6%) (**Figure 8B**). Although ROS induced by the combination of MK-2206 and WZB117 correlated well with increased DNA damage, ROS induction by MK-2206 (79.6%) or WZB117 (3.5%) showed an opposite trend with γ-H2AX induction (**Figures 5B** and **6C**, right panel). Therefore, it is not clear whether ROS is the cause of DNA damage in MDA-MB-231 cells.

## Discussion

MK-2206 is a highly selective and potent allosteric Akt inhibitor and has been shown to enhance the antitumor effect of chemotherapeutic agents (Hirai et al., 2010; Lin et al., 2015). However, it has limited clinical anticancer activity as a single agent (Konopleva et al., 2014; Ahn et al., 2015). Current strategy is to combine MK-2206 with a variety of other anticancer agents. It has been reported that *GLUT1* gene transcription is regulated by activated Akt1, resulting in accumulation of GLUT1 mRNA and protein (Barthel et al., 1999). However, to our knowledge, no evaluation of the combinatorial anticancer effect of Akt inhibitors and GLUT1 inhibitors has been reported yet. Here, we demonstrate for the first time that the combination of MK-2206 and WZB117 synergistically inhibits MCF-7 and MDA-MB-231 cell growth.

The concentration of WZB117 used in most of our mechanism studies was 60 µM, which was higher than the concentration of WZB117 used in A549 non-small cell lung cancer cells (Liu et al., 2012), raising a concern of off-target effects. The IC_50_ of WZB117 in A549 cells was ∼10 µM, and this concentration was used in most of the studies in A549 cells. The IC_50_ of WZB117 in the cell lines used in our studies were within 40–60 µM. It has also been reported that the IC_50_ of WZB117 in MCF-7 cells was 42.66 µM (Chen et al., 2017a), similar to our results. Therefore, 60 µM WZB117 was chosen for mechanism studies. Western blot analysis of some proteins using 3 µM MK-2206- and/or 30 µM WZB117-treated MCF-7 cells and 6 µM MK-2206- and/or 30 µM WZB117-treated MDA-MB-231 cells was conducted and protein profiles observed were similar to those obtained from cells treated with the combination of 6 or 12 µM MK-2206 and 60 µM WZB117 (**Supplementary Figure S2**). Although the possibility of off-target effects could not be excluded, the growth inhibitory effect of WZB117 showed a good correlation with its inhibitory effect on glucose transport (**Figures 1A** and **2A**), and knockdown of GLUT1 also enhanced the growth inhibitory effect of MK-2206 (**Figure 2B**), indicating that inhibition of GLUT1 by WZB117 at least in part accounted for its synergistic effect with MK-2206.

Interestingly, GLUT1 was upregulated by MK-2206 in MCF-7 cells, as illustrated in **Figure 2C**, and this phenomenon may be associated with resistance to MK-2206. It has been reported that GLUT1 is induced in head and neck squamous cell carcinoma cell lines treated with increasing concentrations of cisplatin and overexpression of GLUT1 is associated with resistance to cisplatin (Li et al., 2015). It has also been reported that increased GLUT1 expression and glucose uptake were involved in gefitinib resistance in non-small cell lung cancer cells (Suzuki et al., 2018). Therefore, the combination with WZB117 may prevent resistance to MK-2206 due to induction of GLUT1 and partly explain why WZB117 can enhance the activity of MK-2206.

MK-2206 inhibits phosphorylation of Akt and its downstream targets. Akt inhibition has been widely accepted to result in growth inhibition in cancer cells (Nitulescu et al., 2016). It was reported that WZB117 slightly downregulated Akt and mTOR phosphorylation in A549 cells (Liu et al., 2012). Our results showed that the combination of MK-2206 and WZB117 could effectively suppress Akt phosphorylation and further decrease p-mTOR, p-p70S6K, and p-4E-BP1 levels compared to either single agent alone in MCF-7 cells. The MK-2206 and WZB117 combination inhibited Akt phosphorylation efficiently, but had no significant effect on p-mTOR, p-p70S6K, or p-4E-BP1 levels in the MDA-MB-231 cells (**Figure 4**). These results suggest that Akt downstream targets other than the mTOR signaling pathway or some off-target effects may also contribute to cell death induced by MK-2206 in combination with WZB117 in breast cancer cell lines.

We have reported that MK-2206 can enhance apoptosis induced by paclitaxel and cisplatin in ovarian cancer cells (Lin et al., 2015). Other studies also indicate that MK-2206 enhances apoptosis induced by chemotherapeutic agents in gastric cancer (Jin et al., 2016) and HepG2 hepatocellular carcinoma cells (Jiao et al., 2013). On the other hand, WZB117 was first reported to induce necrosis in A549 cells (Liu et al., 2012), but was later shown to sensitize MCF-7 and MDA-MB-231 cells to radiotherapy through the induction of apoptosis (Zhao et al., 2016). The combination of MK-2206 and WZB117 significantly and synergistically increased Annexin V-positive MCF-7 and MDA-MB-231 cells, indicative of apoptotic induction. The results were confirmed by Western blot analysis of PARP cleavage in both cell lines and caspase-3 cleavage in MDA-MB-231 cells (**Figure 3**). Altogether, these data indicate that the combination of MK-2206 and WZB117 is capable of inducing apoptosis in MCF-7 and MDA-MB-231 cells.

Akt activation plays an important role in DNA damage response and repair (Xu et al., 2012). MK-2206 was previously reported to sensitize the DNA damaging effect of cisplatin and olaparib in ovarian cancer cells (Whicker et al., 2016). Inhibition of glycolysis has been demonstrated to impede DNA repair (Demel et al., 2015; Liu et al., 2015). When cells encounter DNA damage, the histone variant H2AX is phosphorylated to produce γ-H2AX, which is a marker of DSBs. Chk2 can also be phosphorylated at threonine 68 to activate proteins participating in DSB repair (Podhorecka et al., 2010). Data from Western blot analysis and immunostaining showed that the combination of MK-2206 and WZB117 synergistically enhanced γ-H2AX levels and γ-H2AX-positive MCF-7 cells (**Figures 5A** and **6A, B**). In addition, p-Chk2 levels were also significantly increased after combinatorial treatment in MCF-7 cells (**Figure 5A**). γ-H2AX was dramatically induced in MDA-MB-231 cells as well (**Figures 5B** and **6C**), but clear activation of Chk2 was not observed following MK-2206/WZB117 treatment. Instead, we found that p-Chk1 was markedly induced in MDA-MB-231 cells (**Figure 5B**). Since γ-H2AX could be a consequence of DNA fragmentation during apoptosis (Rogakou et al., 2000), the DNA damaging effect of short-term exposure was then evaluated by the comet assay, and the results indicated that the significant DNA damaging effect occurred within 1–6 h (**Figure 5D**). These data suggest that DNA damage induction could be one of the underlying mechanisms leading to the synergistic cytotoxic effect of MK-2206 and WZB117.

HR and NHEJ are two main mechanisms for the repair of DSBs (Ceccaldi et al., 2016). Akt modulates these repair pathways in complex ways (Xu et al., 2012). Akt1 has been reported to repress HR through inducing cytoplasmic retention of Rad51 and BRCA1 (Plo et al., 2008). On the other hand, it has been demonstrated that PI3K/Akt/mTOR inhibitors enhance radiosensitivity of radioresistant prostate cancer cells in part by downregulating proteins involved in HR (such as Rad51) and NHEJ (such as Ku70/Ku80) (Chang et al., 2014). Moreover, MK-2206 causes impairment of NHEJ and increases radiation sensitivity in several radioresistant cancer cell lines (Holler et al., 2016). Elevation of glycolysis has also been demonstrated to activate the NHEJ and HR pathways, thereby facilitating DNA repair and conferring radioresistance in cells (Bhatt et al., 2015). The MK-2206 and WZB117 combination downregulated Rad51 protein levels and nuclear Rad51 foci formation in MCF-7 cells, implicating that the combinatorial treatment may hinder the HR pathway. Ku80, a crucial protein involved in NHEJ, was also downregulated at the protein level in MCF-7 cells by the combinatorial treatment. Rad51 foci formation and Ku80 protein levels were also downregulated by the MK-2206/WZB117 combination in MDA-MB-231 cells, but the effect was not as dramatic as in MCF-7 cells. HR and NHEJ assays revealed that HR and NHEJ were downregulated by MK-2206 either alone or in combination with WZB117 in MCF-7 cells, but the effect on HR was more dramatic (**Figure 7A**). In MDA-MB-231 cells, HR was also inhibited by MK-2206 or MK-2206/WZB117, but to a lesser extent, and surprisingly, NHEJ was enhanced by the MK-2206/WZB117 combination (**Figure 7B**). However, increased NHEJ efficiency in MDA-MB-231 cells may lead to error-prone DNA repair and may consequently lead to accumulation of cells with DNA damage. These findings suggest that HR deficiency may play a more important role in DNA repair defects caused by MK-2206/WZB117 in MCF-7 cells and increased NHEJ could be the underlying mechanism in MDA-MB-231 cells. Taken together, the MK-2206 and WZB117 combination may not only induce DNA damage but also compromise the DNA repair systems.

MK-2206 has been reported to induce ROS levels and potentiate the cytotoxicity of other anticancer agents (Lin et al., 2015; Chen et al., 2017b). ROS may induce DNA damage (Liou and Storz, 2010). On the other hand, DNA damage may also induce ROS through activation of H2AX (Kang et al., 2012). The DCFH-DA assay revealed that the combination of MK-2206 and WZB117 induced ROS production markedly within 30 min, suggesting that ROS could be the cause of DNA damage, at least in MCF-7 cells. The scenario in MDA-MB-231 cells is more difficult to interpret since MK-2206 but not WZB117 alone markedly induced ROS (**Figure 8B**), but DNA damage induction by either agent was similar based on results from the comet assay (**Figure 5D**). Nevertheless, the combination of MK-2206 and WZB117 did induce significantly more ROS as well as DNA damage in MDA-MB-231 cells.

Triple-negative breast cancer cells, such as MDA-MB-231 cells, are usually more resistant to drug treatment. Although the response of MDA-MB-231 cells to either MK-2206 or WZB117 was not as good as MCF-7 cells, the combination of MK-2206 and WZB117 showed a comparable efficacy in both MCF-7 and MDA-MB-231 cells. Future investigation is required to further dissect the underlying mechanism in MDA-MB-231 cells and further testing in more breast cancer cells is warranted.

## Conclusion

Our findings indicate that the combination of MK-2206 and WZB117 may exert a synergistic cytotoxic effect in both ER(+) MCF-7 and triple-negative MDA-MB-231 breast cancer cells *via* Akt inhibition and ROS induction, which may in turn cause DNA damage. This combination may also impair DNA damage repair and ultimately lead to apoptosis in breast cancer cells.

## Data Availability Statement

All datasets for this study are included in the article/**Supplementary Files**.

## Author Contributions

L-CH, J-HG, and S-WL led the research project. Y-LL and H-CW performed the experiments, analyzed the data, and prepared the figures. J-LH provided technical guidance. L-CH wrote the manuscript.

## Funding

This work was supported by the Ministry of Science and Technology in Taiwan (MOST 105-2320-B-002-020-MY3) and by the National Taiwan University.

## Conflict of Interest

The authors declare that the research was conducted in the absence of any commercial or financial relationships that could be construed as a potential conflict of interest.
