# Supplementary material for: The Combination of MK-2206 and WZB117 Exerts a Synergistic Cytotoxic Effect Against Breast Cancer Cells
Source: Front Pharmacol. 2019 Nov 6;10:1311. doi: 10.3389/fphar.2019.01311 (PMC6856645; doi:10.3389/fphar.2019.01311)
Supplement: Supplementary file 1 [file Presentation_1.pptx]

## Slide 1
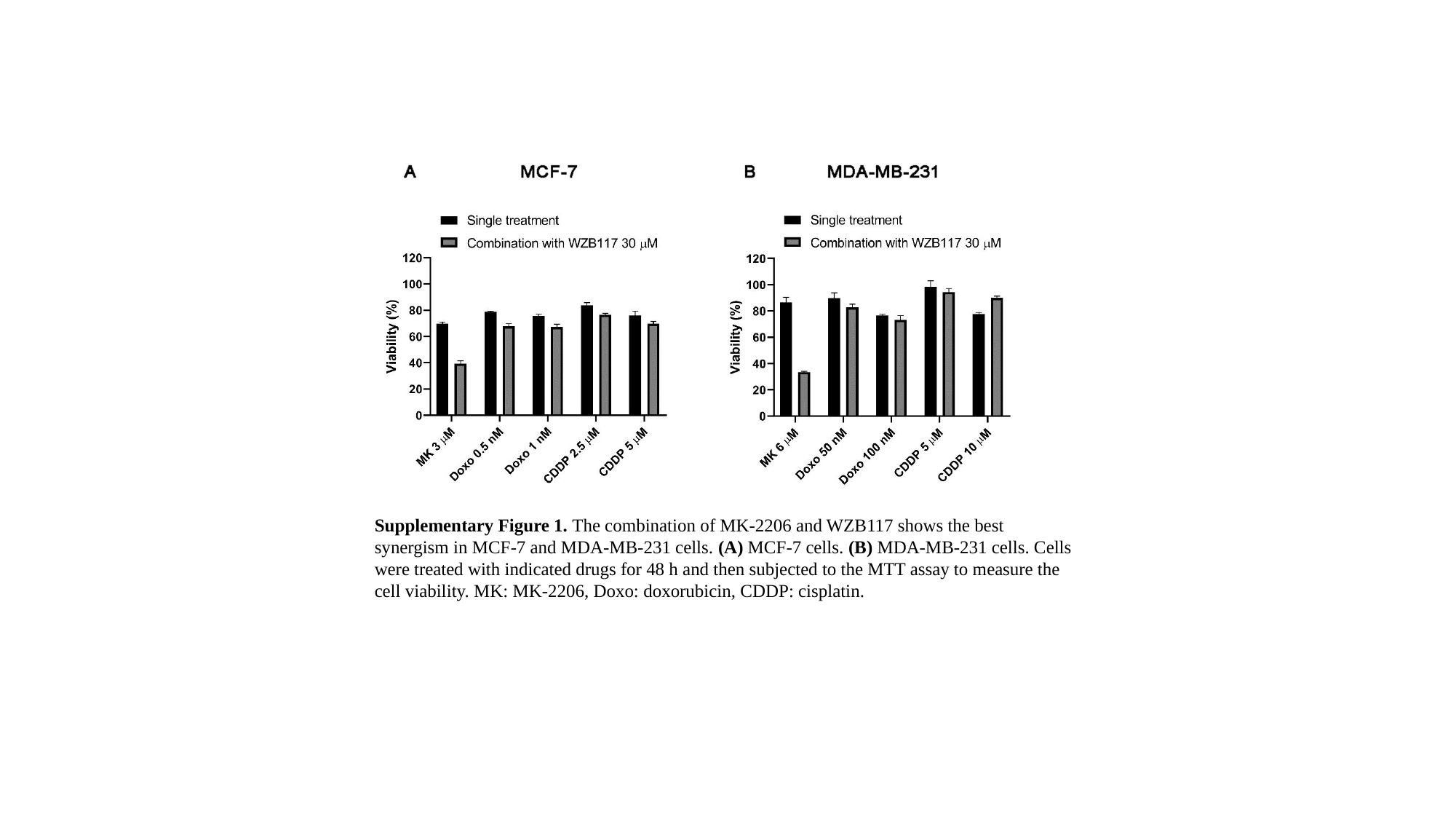

Supplementary Figure 1. The combination of MK-2206 and WZB117 shows the best synergism in MCF-7 and MDA-MB-231 cells. (A) MCF-7 cells. (B) MDA-MB-231 cells. Cells were treated with indicated drugs for 48 h and then subjected to the MTT assay to measure the cell viability. MK: MK-2206, Doxo: doxorubicin, CDDP: cisplatin.

## Slide 2
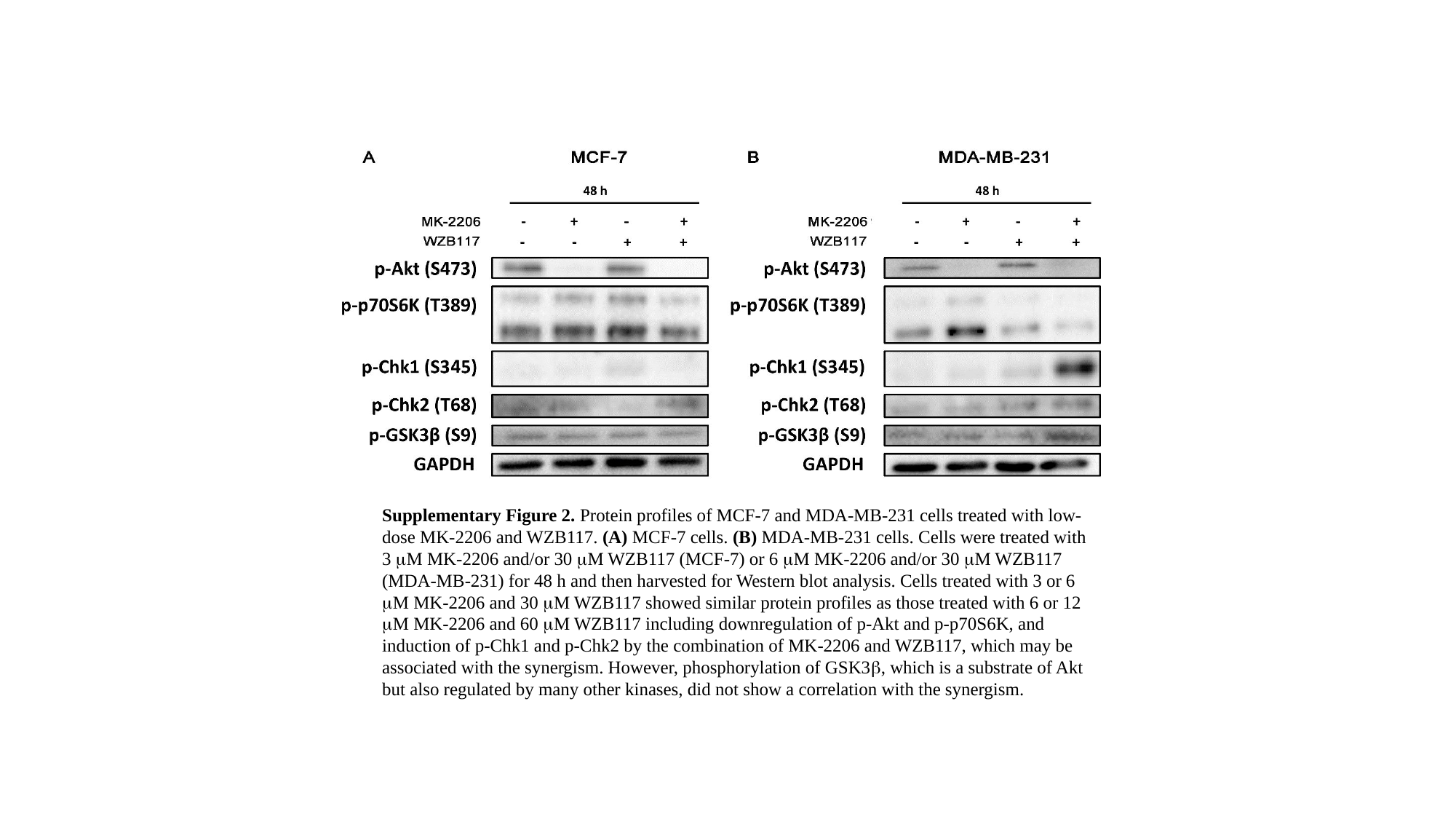

Supplementary Figure 2. Protein profiles of MCF-7 and MDA-MB-231 cells treated with low-dose MK-2206 and WZB117. (A) MCF-7 cells. (B) MDA-MB-231 cells. Cells were treated with 3 mM MK-2206 and/or 30 mM WZB117 (MCF-7) or 6 mM MK-2206 and/or 30 mM WZB117 (MDA-MB-231) for 48 h and then harvested for Western blot analysis. Cells treated with 3 or 6 mM MK-2206 and 30 mM WZB117 showed similar protein profiles as those treated with 6 or 12 mM MK-2206 and 60 mM WZB117 including downregulation of p-Akt and p-p70S6K, and induction of p-Chk1 and p-Chk2 by the combination of MK-2206 and WZB117, which may be associated with the synergism. However, phosphorylation of GSK3b, which is a substrate of Akt but also regulated by many other kinases, did not show a correlation with the synergism.
